# Factors influencing customer satisfaction and loyalty towards urban railway services in Hanoi: Mediating role of perceived value

**DOI:** 10.1371/journal.pone.0340760

**Published:** 2026-01-08

**Authors:** Xuan Can Vuong, Thi Minh Trang Vu, Duc Anh Trieu, Trong Thuat Vu

**Affiliations:** 1 Faculty of Environment and Transport Safety, University of Transport and Communications, Hanoi, Vietnam; 2 Faculty of Electrical-Electronic Engineering, University of Transport and Communications, Hanoi, Vietnam; University of Jaen: Universidad de Jaen, SPAIN

## Abstract

Understanding the satisfaction and loyalty of customers using urban railway services is important because customer loyalty is considered a key determinant of the operational and economic efficiency of this service. The purpose of this study is to explore the relationships between customer satisfaction and loyalty and their influencing factors, including service quality and perceived value. By using data collected from customers of Hanoi’s urban railways, a newly operating public transport system in Vietnam, this study uses confirmatory factor analysis (CFA) and structural equation modeling (SEM) to analyze the structural paths of the conceptual model. The results reveal that relationships are statistically significant and perceived value is an important mediator. The study concludes by drawing out implications that will help service providers, regulators, policymakers, planners, and researchers develop effective strategies to increase the use of urban railway services in Hanoi and other major cities.

## Introduction

Along with the swift urbanization trend and rising travel demand, Hanoi, as a cultural-economic-political center of Vietnam, is facing numerous challenges in the urban road network, such as population, activities, traffic accidents, air pollution, traffic jams, and so on [1]. For instance, according to official statistics, in 2024, the city managed to reduce 13 out of 33 congestion points during peak hours but simultaneously allowed the emergence of 16 new points. Consequently, the overall number of congestion points remained unchanged.

Ha Noi has a population of over 8.5 million people (not including about 1.2–1.5 million people from other provinces and cities who regularly live, work, and study in the capital), but the daily means of transport of the people is still personal vehicles, especially motorbikes (accounting for about 80% of trips). In the context of limited land funds for expanding the road network, the growth rate of personal vehicles shows no signs of slowing down. For example, according to statistics, Hanoi has about 9.2 million vehicles of all kinds in operation. The overall growth rate of vehicles is about 4–5% per year, 11–17 times higher than the speed of road expansion with an average growth rate of 0.3% per year) [2]. Hence, promoting the development of public transport systems, especially mass passenger public transport such as urban railways, encourages people to limit the use of personal vehicles and increase the use of public transport and non-motorized vehicles, which are fundamental solutions to solve traffic problems in Hanoi city.

Hanoi’s urban railway network is planned with 14 routes of a length of 550 km [3]. Up to now, only 2 routes with a length of about 21.6 km have been put into commercial operation, including Line 2A (Cat Linh – Ha Dong) with a length of 13.1 km and the first section of Line 3 (Nhon to Cau Giay) with a length of 8.5 km. Those lines are managed and operated by Hanoi Metro Company (HMC) [4]. Line 2A runs entirely elevated through 12 stations and has been commercially operating since November 6, 2021. Line 3 also has 12 stations, but the first section, with a length of 8.5 km, runs above ground, and the remaining section, with a length of 4 km, runs underground. The first section has been commercially operating since August 8, 2024 [4]. Thus, the urban railway is a new transportation system in Hanoi City. The first two routes put into operation not only have significance in terms of urban transport infrastructure but also are a pilot step in the process of transforming urban transport models towards modernity and environmental friendliness.

According to HMC data, Hanoi’s urban railway system carried about 8 million passengers in the first five months of 2025, including over 5.4 million on Line 2A (a 15% increase over the same time in 2024) and over 2.5 million on the first sectiont of Line 3. While the initial segment of Line 3 had an average of 19–21 thousand passengers per day, Line 2A had an average of 40–45 thousand passengers per day. As a result, they have made a substantial contribution to enhancing Hanoi’s public transportation system’s effectiveness, easing traffic jams, lowering pollution levels, and fostering socioeconomic growth.

Although Hanoi’s urban railway network has just been put into operation, it has attracted a significant number of people who choose to travel by personal vehicle. The more personal vehicles switch to urban railways, the more benefits there will be for traffic congestion. In fact, satisfaction with the quality of urban railway services is an aspect that clearly affects users’ choices. Customers who have a satisfactory experience with the service provided are likely to use the service again, while customers who have problems with the service provided are likely not to use public transport services next time. Customer satisfaction is likely to maintain their loyalty in a competitive environment among transport modes. Satisfied customers will tell others about their experience [5], thereby increasing the appeal of the service, enhancing the company’s image, etc.

To measure customer satisfaction and loyalty in the urban railway field, many studies have been conducted so far, but it is still difficult to draw a common model. Therefore, researchers are still trying to understand the main factors affecting customer satisfaction and loyalty in different specific contexts of urban railway fields. Additionally, most of the existing literature related to satisfaction in this field is mainly focused on developed countries. There are only a few studies on satisfaction and its influencing factors in the context of developing countries like Vietnam.

So far, many studies have focused on service quality and perceived value as the key factors in the development of satisfaction and loyalty of customers in the field of urban railways. However, despite the increasing interest, there is still a lack of consensus on some aspects, for example, the mediating effect of perceived value between service quality and satisfaction, or the mediating effect of satisfaction between perceived value and loyalty, or the mediating effect of perceived value between service quality and loyalty, or the direct effect of perceived value on loyalty, etc.

Hence, the main purpose of this study is to identify the factors, including service quality and perceived value, that influence and evaluate the level of customer satisfaction and loyalty with the quality of urban railway services provided by Hanoi. At the same time, this study will consider the mediating role of perceived value in the relationships among service quality, customer satisfaction, and loyalty. Furthermore, this study hopes to contribute to the formation and development of a tool to measure customer satisfaction and loyalty through a chain of service quality – perceived value – satisfaction – loyalty.

### Literature review

This section examines important research on a paradigm of service quality – perceived value – satisfaction – loyalty in urban railways. Customer satisfaction is an emotional state in which a product or service meets or exceeds the customer’s demands and expectations [6]. It originates from personal needs, previous experiences, and external information, such as advertising, word of mouth from friends, family, and so on. Therefore, it is often used as an important measure of how well the products and services provided by a company meet or exceed customer expectations. It has a direct impact on the business success of a service-providing company, reflecting the customer’s willingness to continue using and recommend a product or service, i.e., consumer behavior intentions or customer loyalty [7,8]. The idea of perceived value is established in Fornell’s model [5]. It is the customer’s overall assessment of a product or service after taking perceived quality and price into account. So, the customer’s economic assessment of the services that they utilize is known as perceived value.

It has been demonstrated that customer satisfaction and loyalty are significantly predicted by several factors, such as service quality [9] and perceived value [5,10,11]. Zeithaml defined service quality as the customer’s evaluation of the overall superiority or excellence of the service [12]. The most well-known scales for measuring service quality include SERVQUAL [13], IPA (Importance-Performance Analysis) [14], SERVPERF [15], and others. Given the intricacy of the concept, the ambiguity around the attributes employed, and the diversity of consumer opinions, measuring service quality poses many challenges in research, leading to the use of different scales to measure it [16]. The most widely used scale for measuring and monitoring service quality among them is the SERVQUAL scale proposed by Parasuraman et al. In the SERVQUAL model, service quality, as the discrepancy between consumers’ perceptions and expectations, is measured based on five important factors, including reliability, assurance, tangibles, responsiveness, and empathy [13]. Although the SERVQUAL scale is a solid foundation for many studies, it has certain limitations that need to be further expanded and adapted to different contexts. For instance, the SERVPERF model proposed by Cronin and Taylor is an improvement of the SERVQUAL model by removing the observed items related to expectations and measuring only the performance items; Cavana and Corbett [17] extended the original SERVQUAL with three new dimensions, including comfort, connection, and convenience, to evaluate service quality of passenger railways in Wellington, New Zealand. They showed that the instrument’s reliability is supported by high Cronbach alpha values; Ma improved the SERVQUAL scale with two new dimensions (intelligence and connectivity) that consider customers’ actual perceptions of metro transport service quality [9]; Zhuang et al. [18] used the SERVQUAL to identify its main factors affecting metro service quality of first-tier cities in China. Irfan et al. [19] modified SERVQUAL with three new dimensions (i.e., safety, information, and food) to measure the perceptions of customers towards the railway service quality in Pakistan.

To date, many models have been developed to measure customer satisfaction and its relationship with influencing factors, such as ACSI [5,10], ECSI [11], the technical and functional quality model, the model of Oliver [20], antecedent and mediator model [21], etc., The model of antecedents and mediators proposed by Dabholkar et al. [21] is shown that the antecedents of service quality, including reliability, personal attention, comfort, and uniqueness, will influence customer satisfaction and consumer behavior intentions [21]. Fornell et al. showed that the factors affecting customer satisfaction and loyalty in different industries are not uniform [5]. In general, different models approach customer satisfaction from different perspectives. However, they all agree on one point that the antecedents of service quality will lead to customer satisfaction or dissatisfaction.

Regarding the relationship between service quality with customer satisfaction and loyalty, some studies have suggested that service quality is one of the antecedents of customer satisfaction and loyalty. Most of them considered the direct effect of service quality on customer satisfaction [7,9,18,22], and the indirect effect of service quality on customer loyalty through customer satisfaction [7,9,18]. For example, Cronin and Taylor argued that customer satisfaction can directly influence customer loyalty, and service quality is an important antecedent of satisfaction [15]. A few studies considered the indirect effect of service quality on customer satisfaction and loyalty through intermediate factors, such as perceived value. However, numerous studies agreed that better customer satisfaction and loyalty are correlated with high-quality services. In other words, increasing customer satisfaction and loyalty can be facilitated by improving service quality [13,15]. For relationship perceived value and customer satisfaction and loyalty, several studies have shown that perceived value has a direct positive impact on customer satisfaction in many service industries with different research models, such as ACSI [5,10], ECSI [11]. Besides, customer satisfaction has also been found to be a mediating factor between perceived value and customer loyalty [5,10,11,23]. As a result, perceived value is a significant antecedent factor that explains customer satisfaction and loyalty.

Like other fields, in the field of transportation (such as bus systems, urban railway systems, transit services, payment services, etc.), determining the influencing factors as well as assessing the level of satisfaction and loyalty are common and continuous research topics to serve as a basis for building a suitable transportation system that meets people’s travel needs. There have been many and varied studies on this topic. Some instances from urban railway services suggest that several factors may influence customer satisfaction and loyalty through the results of the SEM approach, such as Stuart et al. [24] used SEM to assess customer satisfaction for the subway system of New York City. They found that some factors had a direct influence on satisfaction, while others had an impact through mediating variables (e.g., security, speed, and value). There were also differences in the weights of the influences of different factors; Lai and Chen emphasized via path analysis that service quality, perceived value, and satisfaction are important factors when assessing the causal relationship between involvement and passenger behavioral intentions on KMRT in Taiwan [8]; Saw et al. revealed through principal component analysis that three factors (security, safety, and comfort; ticket purchase facilities; infrastructure quality) were capable of predicting passenger satisfaction on Tyne and Wear Metro, UK [25]. Eboli and Mazzulla used a SEM to examine how passengers perceive train services in Northern Italy and discovered that cleanliness, regularity and frequency of runs, and punctuality had the biggest beneficial effects on service quality [26]; Ibrahim et al. applied the SEM to establish the factors influencing customers’ satisfaction with the services of the light rail transit in Kuala Lumpur, Malaysia [27]. They proved that perceived quality and perceived value have direct effects on customer satisfaction. Shen et al. used SEM and the ACSI model to reveal a direct and positive correlation between the perceived value and passenger satisfaction on the system of Suzhou rail transit [28]; De Oña et al. [29] used the SEM approach to examine the relationship between perceptions, satisfaction, and intentions of passengers with the Metro of Seville, Spain. They found that these two passenger groups have somewhat different attitudes towards the service of light rail transit. Concurrently, their research results also supported the service quality – satisfaction – loyalty paradigm; Yilmaz et al. [23] applied the ACSI model and SEM to measure service quality of the light rail transit in Eskisehir of Turkey. They found that perceived value acted as a mediator between perceived quality and customer satisfaction; Ma [30] established a model to evaluate satisfaction through service quality and perceived value. He revealed that perceived value plays a mediating role between service quality and satisfaction. However, Ma’s model did not consider customer loyalty; Zhuang et al. [18] applied a paradigm of service quality – satisfaction – loyalty and SEM to assess subway service quality in some cities in China. They found that the empathy factor of service quality has no significant impact on satisfaction; De Ona et al. [22] used SEM to examine the mediating role of customer satisfaction in the relationship between service quality and customer loyalty corresponding to different models in public transport service in several European cities. They revealed that the full mediation model of customer satisfaction is superior to its partial mediation model. These studies show that the study results are diverse but not unified, depending on the type of service, time, location, scale, and application models. Besides, very few studies consider the role of mediating factors, such as perceived value in a chain of service quality – perceived value – satisfaction – loyalty applied in the urban railway services. Therefore, research on satisfaction and factors affecting it, such as service quality and perceived value, should be further studied during the exploitation process of urban railways corresponding to each specific context.

In Vietnam, studies on customer satisfaction or influencing factors in the transportation sector in general and urban railways in particular are still relatively rare. These studies can be hard to find because of a lack of public disclosure or a limited, internal research scope, which leaves researchers without enough data to carry out in-depth research and validate findings. Through the research process, this study has collected several studies. Typically, Pham et al. (2024) looked at the effects of social and environmental elements on customer satisfaction with urban railways [31]. They found that customer satisfaction is positively impacted by the conduct, demeanor, and surroundings of employees. However, the study’s scope is limited, and only a small number of factors were evaluated; Nguyen-Phuoc et al. (2020) [32] examined factors influencing the satisfaction and loyalty of ride-hailing passengers in Vietnam. They revealed that perceived service quality, perceived sales promotion, and perceived benefits of the booking app had a direct impact on satisfaction and loyalty. However, the number of factors considered in the study is very modest, only 3 factors related to perceived quality. Moreover, the interactions between these factors have not been considered; Huong Ly et al. (2025) [33] assessed how satisfied senior citizens were with bus travel in the setting of free fares. They discovered that one element that significantly affected passenger happiness was the free fare policy. However, older age groups and women tended to be happier with bus services; Tran et al. (2025) [34] analyzed the satisfaction of passengers with the services of long-distance bus stations in Vietnam. They found that safety and security, comfort, reliability, customer service, and information availability all had a positive correlation with satisfaction.

From the above analysis, the main aim of this study is to measure service quality and perceived value in Hanoi’s urban railway services and their impact on the satisfaction and loyalty of customers.

## Methods and materials

### Research framework and hypotheses

**Fig 1:**
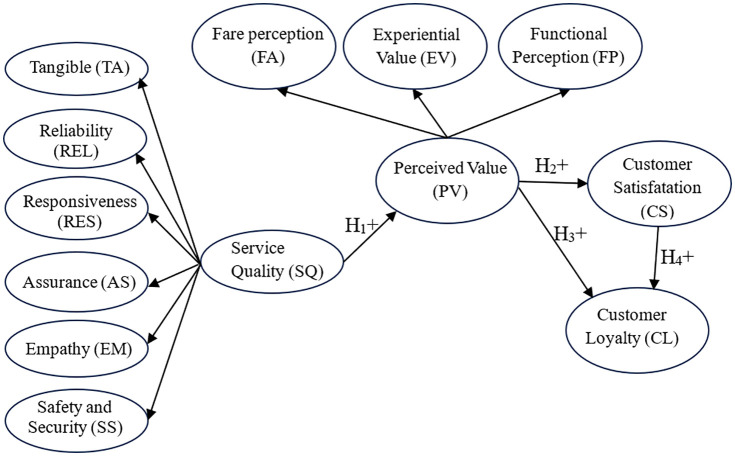
Research framework.

A research framework, as shown in Fig 1, is suggested following a review and synthesis of the relevant research models and background theories published in esteemed domestic and international journals. It is also founded on the antecedent and mediator conception in the work of Dabholkar et al. [21], the “indirect model” concept in Cronin et al. [7], and a chain of service quality -perceived value- satisfaction in Lai and Chen [8] and Ma [9]. Hence, in this study, service quality is an antecedent, the perceived value is a mediating factor, and customer satisfaction and loyalty are outcomes in the research framework as a chain of service quality – perceived value – satisfaction – loyalty.

In the research framework, service quality (SQ) reflects the degree to which a product or service meets predetermined customer requirements, and suppliers must periodically review these quality requirements [13]. Therefore, the service or product can match, meet, or exceed the consumer’s needs. Currently, the scales of SQ are also diverse and inconsistent. For example, in the original SERVQUAL model, service quality is originally measured through ten sub-factors [35], which is later reduced to five sub-factors, respectively called tangible (TA), reliability (REL), responsiveness (RES), assurance (AS), and empathy (EM) [13]; Saw et al. [25] explored three factors (i.e., security, safety, and comfort; ticket purchase facilities; and infrastructure quality) in SQ; Eight dimensions (i.e., TA, accessibility, availability, information, security, individual space, customer service, and environmental pollution) were used to measure SQ in the research of De Oña et al. [29]; Seven factors, which included TA, EM, AS, REL, RES, intelligence, and connectivity, have been compressed into SQ in the study of Ma [30]; Lai and Chen [8] only considered two dimensions of SQ (core service and physical environment); Model of Cronin and Taylor only used one dimension of perceive performance to describe SQ [15]; etc. In this study, to determine the key factors inﬂuencing the customers’ satisfaction with Hanoi’s urban railway services, SQ consists of six sub-factors (i.e., TA, REL, RES, AS, EM, and SS), which are adjusted and expanded from observation items of the SERVQUAL model suitable for Hanoi. Compared with the dimensions of the five-factor SERVQUAL, this study adds the safety and security (SS) factor. This is also the factor mentioned in the original 10-factor version of SERVQUAL and has recently been considered by some studies as an important factor of SQ [34]. The benefits that consumers receive are a reflection of perceived value (PV). Value is like perceived cheap cost, value obtained relative to cost, desired satisfaction, and obtained advantages [36]. Customer perception of service best reflects service quality. Up to now, many perspectives on measuring perceived value have been proposed. For example, according to Zeithaml et al. [12], PV comprises two main components (i.e., get/benefits and give/sacrifices); In the study of Ledden et al. [37], PV has eight sub-factors, named respectively functional, epistemic, emotional, image, social-others, social-students, time, and money; Sweeney and Soutar consider that PV includes functional value, social value, emotional value, price-quality correlation perception [38]; Koller et al. suggest that PV consists of functional value, social value, economic value, emotional value, and ecological value [39]; PV in the study of Ma [30] includes functional value, cost value, and experience value; Shen et al. [28] also measured QV by one dimension of two observed variables; Several studied only examined one dimension of the observed variables related to QV in their research model, such as Ibrahim et al. [27], Yilmaz et al. [23]. Although the number of dimensions and the number of observed variables were different, most studies have agreed on the positive influence of PV on CS. On the basis of the qualitative research results and referring to the above related studies, this study examines PV with three sub-factors, including functional perception (FP), experiential value (EV), and fare perception (FA). Based on several previous studies, it was found that SQ has a positive and significant impact on PV. Hence, the following hypothesis is proposed:

*Hypothesis H1: Service quality has a positive impact on perceived value (H1).*

Customer satisfaction (CS) can be described as the fulfillment of customers’ needs and expectations for a particular product or service they consume [40]. According to Parasuraman et al. [13], CS reflects the correlation between expectations and perceived value from a product or service. When a product or service meets their pre-determined expectations, they are satisfied and fulfilled. Up to now, most studies use one dimension of observed variables to measure CS. Similarly, this study also considers one dimension to measure CS.

Customer loyalty (CL) is considered the feeling of attachment and affection towards a product or service in the future. In addition, CL also includes the willingness to share and recommend that product or service to others. Until now, most studies have used one dimension of observed variables to measure CL. Accordingly, this study also considered one factor of observed variables to measure CL. So far, some studies have been conducted and found that PV has a positive inﬂuence on CS and CL. For example, Shen et al. [28] found that there is a direct positive link between perceived value and satisfaction in the rail transit system of Suzhou, China. As a result, the following hypotheses are proposed:

*Hypothesis H2: Perceived value has a positive impact on customer satisfaction (H2).*

*Hypothesis H3: Perceived value has a positive impact on customer loyalty (H3).*

In customer loyalty (CL), one of the most widely used attitudinal metrics is customer satisfaction (CS). Several studies have demonstrated that the positive influence of CS on CL [32]. In the urban railway context, CS is considered a major direct factor influencing CL [10]. Therefore, the next hypothesis can be formulated:

*Hypothesis H4: Customer satisfaction has a positive impact on customer loyalty (H4).*

### Data collection

To match the research framework, a self-reported questionnaire was designed to interview participants who were passengers of urban railway services in Hanoi, the capital of Vietnam. The questionnaire included two sections. The first section contained demographic profiles of participants, such as their age, gender, purpose, and frequency of trips. The second section was the items related to service quality, perceived value, customer satisfaction (CS), and customer loyalty (CL). Among them, service quality had six sub-scales adjusted from SERVQUAL models [13], including tangible (TA), reliability (REL), responsiveness (RES), assurance (AS), empathy (EM), safety and security (SS); perceived value had three sub-scales (i.e., functional perception (FP), experiential value (EV), and fare perception (FA)) adjusted from other studies related to satisfaction, as shown in Table 1. All the rated items of the second section were measured on a five-point Likert scale (from “1” =”strongly disagree/very dissatisfied” to “5” = “strongly agree/very satisfied”). After adjustment and supplementation, the scales of the second part become the official scale presented in Table 1 as follows:

**Table 1:** The constructs and items of the research framework.

Construct	Item	Source
**Tangible (TA)**	Modern equipment with clear information (TA1)	[13], [8,26,33,37]
	Staff dressed politely and professionally (TA2)	[36]
	Staff are always friendly and welcoming to customers (TA3)	[30]
	Clean environment at stations and on trains (TA4)	[8], [25,26,28–30,33]
	Accessibility for disadvantaged customers (TA5)	
	Adequate parking areas around stations (TA6)	[26]
**Reliability (REL)**	Trains run on time according to schedule (REL1)	[34], [33,36,37]
	Provision of train schedule information (REL2)	
	Update correct trip information at stations (REL3)	
	Prior notification of changing schedule (REL4)	[34]
	Simple and reliable ticketing purchasing process (REL5)	
**Responsiveness (RES)**	Clear announcements when trains arrive (RES1)	[36]
	Staff can handle various situations (RES2)	[13], [30,36,37]
	Provide prompt service (RES3)	
	Willingness to assist customers (RES4)	[13], [36,37]
**Assurance (AS)**	Staff make customers feel comfortable (AS1)	[36], [37]
	Ensure to provide information when trains are delayed and other information during the journey (AS2)	
	Ensure customers are safely transported to their destination (AS3)	[30]
**Empathy (EM)**	Train schedule meets customer needs (EM1)	[33]
	Understanding customer needs (EM2)	[13]
	Prioritizing customer interests (EM3)	[36]
	Ready to assist customers on trains and at stations (EM4)	[36], [37]
**Safety and security (SS)**	No concerns about staff or customer rudeness (SS1)	[25], [29,34]
	No concerns about disorder at stations and on trains (SS2)	[25], [29,34]
	Feeling safer using the urban railway (SS3)	[8], [26,28,29,34]
**Functional perception (FP)**	Convenient inquiries about operations and tickets (FP1)	[30]
	Comprehensive station services (FP2)	[39]
	Clear signage and directions make it easy to find the information customers need (FP3)	[30]
	Easy movement in and out of stations (FP4)	[30]
**Experiential value (EV)**	Excellent service, very impressive (EV1)	[30], [38]
	The environment on the train and at the station felt comfortable and pleasant (EV2)	[30], [38,39]
	Trips always make me happy (EV3)	[30], [38,39]
**Fare perception (FA)**	Service justifies the fare (FA1)	[27], [37–39]
	Competitive price (FA2)	[38], [39]
	Fare meets expectations (FA3)	[37]
**Customer loyalty (CL)**	Willing to introduce the advantages of urban railway to acquaintances (CL1)	[30], [32]
	Encourage family and friends to use the service (CL2)	[28], [23,30,32],
	Willing to continue using the service (CL3)	[30], [32]
	I would still use it without discounts (CL4)	
**Customer satisfaction (CS)**	Satisfied with overall service quality (CS1)	[27], [23,28,32]
	Satisfied with staff attitude (CS2)	
	Satisfied with fares (CS3)	
	Satisfied with support (CS4)	
	Satisfied with the facilities on board and at the station (CS5)	

To analyze the relationship between CS, CL, and influencing factors through SEM, a sufficiently large sample size is required [41,42]. When applying the Maximum Likelihood estimation method, a sample size greater than or equal to 200 is considered a large sample [43]. In this study, convenience sampling was used to choose the sample at Hanoi’s urban railway stations, such as Line 2A and the first section of Line 3. From May 20 to June 30, 2025, passengers were interviewed face-to-face as they were waiting to board or just coming off the train at periods of steady passenger volume, which amply demonstrated their service consumption patterns.

Regarding ethical approval, this study was evaluated and given ethical approval by the Operating Enterprise of Urban Railway Line No. 2A (Document No. 139.1/CV-XN2A) and the Operating Enterprise of Urban Railway Line 3 under Hanoi Metro Company (Document No. 152.1/CV-XN3). These enterprises confirmed that this study posed little risk to the participants and complied with the administrative procedures of the enterprises, the data collected was completely anonymous, so the participants’ verbal consent was appropriate, and their written signature was not required.

During the implementation period from May 20 to June 30, 2025, consent to participate in the study was obtained and recorded directly by the investigator at the time of approaching potential participants. Before the survey, the surveyor provided complete information regarding the research objectives, survey content, the voluntary nature of participation, and the right to refuse or withdraw from the study at any time, and informed participants that the study had been formally approved in documents by the Operation Enterprises of Urban Railway Lines. Only participants who verbally expressed their consent were included in the survey. Participants who declined to participate or discontinued participation were not recorded and were not included in the study sample. No witnesses or written consent with signatures were required, as the study was conducted anonymously, did not collect personally identifiable information, and involved minimal risk.

### Data analysis techniques

Questionnaire data are coded and entered using SPSS 25. Data analysis is performed on SPSS 25 [44] and AMOS 24 [45]. The analytical framework is evaluated by two stages, including the measurement model and the structural model [46]. The former evaluates the reliability and validity of the constructs in the framework through confirmatory factor analysis (CFA) in order to verify the factor structure of the items and ensure the accuracy of the measured constructs. The latter examines the path coefficients in an analytical framework and understands the relationships between the constructs through SEM [47]. It is one of the most popular approaches because it can handle a lot of endogenous and exogenous variables [48].

Before performing the SEM model, composite reliability (CR) and Cronbach’s Alpha (CA), which gauge the scales’ internal consistency, are used to analyze reliability testing. CA ranges from 0.6 to 1.0 to ensure consistency between items in the same scale. CR of the scale is greater than 0.7, proving that the scale is reliable. Simultaneously, convergent validity and discriminant validity are used to evaluate validity. Whereas discriminant validity verifies that constructs are different from one another, convergent validity guarantees that elements within a construct are correlated [49]. In this study, convergent validity is assessed through Average Variance Extracted (AVE), while discriminant validity of the scale is examined through the Fornell-Larcker criterion. When AVE is greater than 0.5, the scale achieves convergence.

## Analysis results

### Demographic profiles of participants

With 477 valid responses recorded, the survey sample was analyzed using SPSS 25.0. The distribution was fairly equal by gender, with 51.9% of the population being female and 48.1% being male. According to age categories, the poll was primarily completed by young people, with the majority of respondents (40.0%) being under 25 and the next largest percentage (38.8%) being between 25 and 35. For education level, 55.1% of participants had an undergraduate or graduate degree, indicating that they were a well-educated population. The distribution of marital status was fairly balanced, with 45.7% of people married and 54.3% of people single.

### Measurement model

The analysis results showed that the CA values of the constructs ranged from 0.877 to 0.951, and were all greater than 0.7 [50], meeting the requirements of reliability. Besides, the corrected item-total correlation values of the items in the constructs ranged from 0.691 to 0.889, surpassing the acceptable threshold of 0.5 [50], showing great internal consistency.

The SQ construct has six sub-factors, including TA, REL, RES, AS, EM, and SS. The CFA results of SQ with the maximum likelihood method revealed that the Chi-square = 857.317; df (degrees of freedom) = 260; Chi-square/df (the chi-square to degrees of freedom)= 3.297 < 5 [51,52]; CFI (Comparative Fit Index)= 0.957 > 0.9; TLI (Tucker-Lewis Index) = 0.950 > 0.9 [53]; RMSEA (Root Mean Square Error Approximation) = 0.069 < 0.08 [51]; GFI (Goodness of Fit Index) = 0.875, has almost reached 0.9. Most values of the indexes met the requirements. The measurement model of SQ had a relatively high level of suitability, achieved data conformity, and was reliable. Additionally, the CFA results also showed that the CR values of the factors ranged from 0.93 to 0.95, greater than 0.7 [54,55]. The average variance extracted (AVE) values of the factors ranged from 0.69 to 0.83, greater than 0.5 [50]; Factor loadings of the items ranged from 0.710 to 0.936, greater than 0.5 [49]. Besides, the CR value and the AVE value of SQ also met the requirements, as shown in Table 2, showing satisfactory reliability of SQ, ensuring convergency and the ability to measure related factors of SQ [48,52]. Simultaneously, most of MSV (Maximum Shared Variance) <AVE (except TA, but the difference is not large), and the square root of AVE for each construct in SQ exceeded the correlations with other constructs, as shown in Table 3 [49,55]. Hence, all constructs of SQ have been viewed as acceptable in terms of discrimination.

**Table 2:** Results of CFA.

Construct	CA	AVE	CR	MSV	Notes
**Tangible (TA)**	0.930	0.69	0.93	0.73	
**Reliability (REL)**	0.944	0.77	0.94	0.74	
**Responsiveness (RES)**	0.949	0.82	0.95	0.79	
**Assurance (AS)**	0.937	0.83	0.94	0.79	
**Empathy (EM)**	0.942	0.80	0.94	0.76	
**Safety and security (SS)**	0.924	0.81	0.93	0.76	
**Functional perception (FP)**	0.939	0.78	0.94	0.73	
**Experiential value (EV)**	0.934	0.83	0.94	0.73	
**Fare perception (FA)**	0.877	0.71	0.88	0.69	
**Customer satisfaction (CS)**	0.951	0.78	0.95	–	Only one factor
**Customer loyalty (CL)**	0.935	0.76	0.93	–	Only one factor
**Service quality (SQ)**	–	0.82	0.97	–	Second-order latent variable
**Perceived value (PV)**	–	0.82	0.93	–	Second-order latent variable

**Table 3:** Fornell-Larcker criterion in SQ.

	REL	RES	EM	TA	AS	SS
**Reliability (REL)**	0.879					
**Responsiveness (RES)**	0.852	0.907				
**Empathy (EM)**	0.777	0.801	0.896			
**Tangible (TA)**	0.848	0.838	0.780	0.833		
**Assurance (AS)**	0.860	0.889	0.812	0.857	0.913	
**Safety and security (SS)**	0.752	0.784	0.873	0.789	0.821	0.897

The QV construct includes three sub-factors, namely FP, EV, and FA. The CFA results of QV with the maximum likelihood method revealed that the Chi-square = 99.171, df = 30, Chi-square/df = 3.306 < 5 [51,52], CFI = 0.985 > 0.9, TLI = 0.978 > 0.9; RMSEA = 0.070 ≤ 0.08 [51]; GFI = 0.960 > 0.9. All values of the indices were larger than 0.9 [53], meeting the requirements. The measurement model of QV had a relatively high level of suitability, achieved data conformity, and was reliable. Furthermore, the CR values of the factors ranged from 0.88 to 0.94, exceeding the minimum threshold value of 0.7 [54,55]. The AVE values of the factors ranged from 0.71 to 0.83, greater than 0.5 [50]. Factor loadings of the items ranged from 0.739 to 0.929, exceeding the threshold value of 0.50 [49]. Besides, the CR value and the AVE value of QV also met the requirements, as shown in Table 2, demonstrating the high reliability of QV, confirming convergent validity, discriminant validity, and the ability to measure related factors of QV [48,52]. At the same time, MSV < AVE and the square root of AVE for each construct in QV exceeded the correlations with other constructs, as shown in Table 4 [49,55]. Therefore, all constructs of QV have achieved discriminant validity.

**Table 4:** Fornell-Larcker criterion in QV.

	FP	EV	FA
**Functional perception (FP)**	0.885		
**Experiential value (EV)**	0.851	0.911	
**Fare perception (FA)**	0.833	0.78	0.842

### Structural model

**Fig 2:**
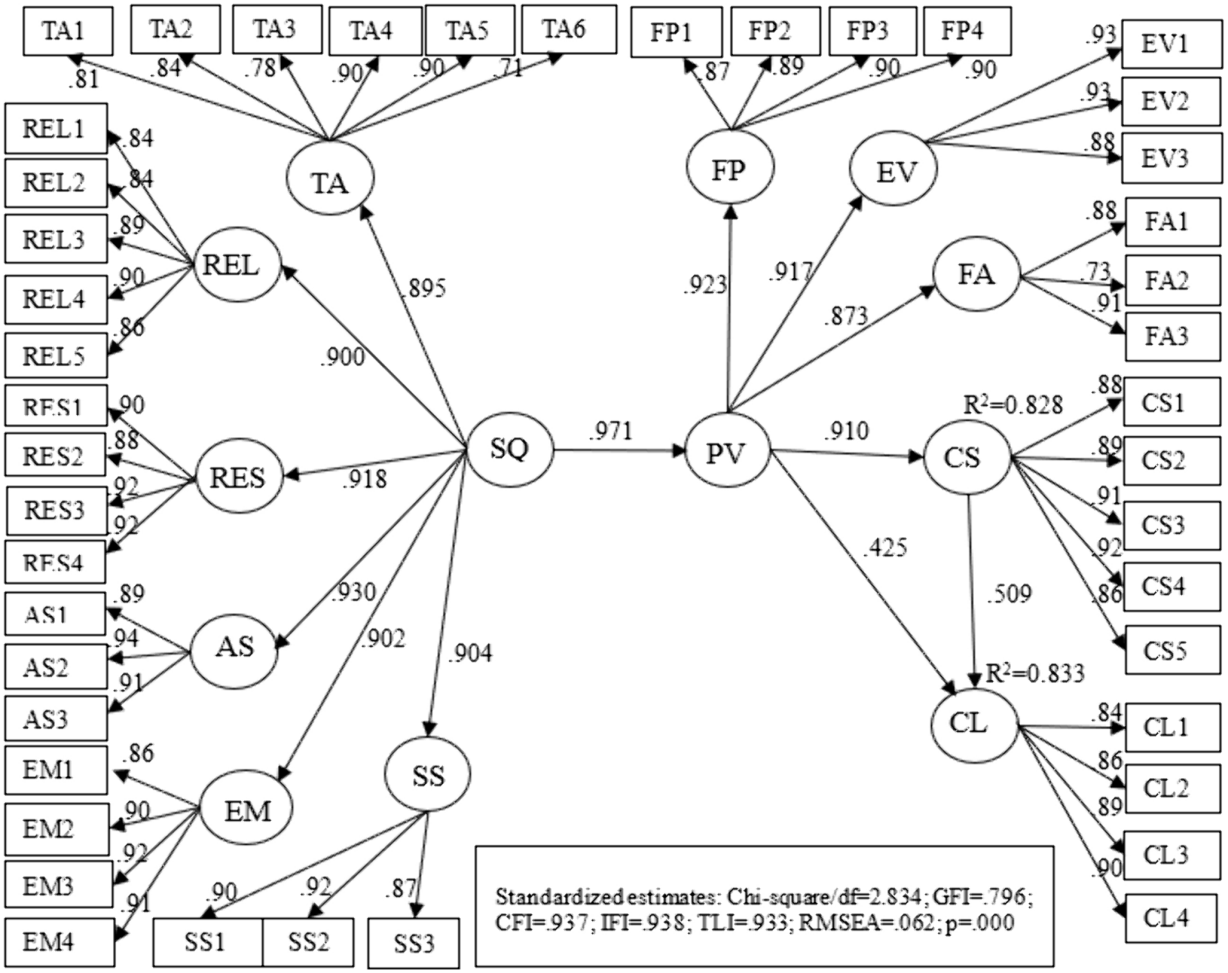
Results of the structural model.

This study tested the analytical framework using SEM to analyze the factors that affect customer satisfaction (CS) and customer loyalty (CL). In the analysis of the model, the goodness-of-fit statistics met the requirements, as shown in Fig 2. The model had the value of Chi-square/df = 2.834 < 5 [51,52]; RMSEA = 0.062 < 0.08 [51]; GFI = 0.796, CFI = 0.937, TLI = 0.933; most indexes were larger than 0.9 (excepting GFI) [53]. Compared with other indexes, GFI is sensitive and lacks sophistication, so it cannot be used as a standalone index [53]. In this study, GFI had not reached the threshold of 0.9, but other indexes still met the requirements [48,56], indicating that the model had a reasonable fit. In the model, all paths were supported with a significance of 0.001, as shown in Table 5.

**Table 5:** Results of the direct effect.

Hypothesis	Path	Standardized estimate	S.E.	C.R.	p	Result
**H1**	SQ → PV	0.971	0.047	21.465	***	Supported
**H2**	PV → CS	0.91	0.041	21.858	***	Supported
**H3**	PV → CL	0.425	0.077	5.602	***	Supported
**H4**	CS → CL	0.509	0.078	6.714	***	Supported

Notes: “***” corresponding to a significance level less than 0.001.

From the results in Table 6, it is shown that the indirect effects on CS and CL all have positive coefficients. Simultaneously, it confirmed the partial mediating role of PV and CS.

**Table 6:** Results of the indirect effect.

	SQ	PV
**Customer satisfaction (CS)**	0.884	0
**Customer loyalty (CL)**	0.863	0.463

From the results in Tables 5 and 6, it is easy to obtain the total effect of the factors on CS and CL as shown in Table 7.

**Table 7:** Results of the total effect.

	SQ	PV	CS
**Perceived value (PV)**	0.971	0	0
**Customer satisfaction (CS)**	0.884	0.91	0
**Customer loyalty CL)**	0.863	0.888	0.509

Taking into account both direct and indirect impacts on CL, it was found that PV (standardized estimate of 0.888) had the most influence, followed by SQ (standardized estimate of 0.863) and CS (standardized estimate of 0.509). The results showed that, in terms of direct impact, CS has a greater impact on CL than PV; however, when considering indirect impact, PV has a greater impact on CL than CS. Here, CS has played an intermediary role in the relationship between PV and CL, increasing the influence of PV on CL. This means that CS plays an important role in fostering customer loyalty. It is not only a direct factor affecting loyalty but also an intermediary factor for the impact of other factors. In addition, the results also showed that PV has an important role in strengthening CS and fostering CL in urban railway services. PV not only directly affects CS and CL but also plays an important intermediary role in the impact of SQ. Finally, the analytical framework had contributed to 82.8 percent of the variance in CS (R-squared of 0.828) and 83.3 percent of the variance in CL (R-squared of 0.833), considering acceptable [57].

This study used a personal survey questionnaire, so the possibility of common method bias (CMB) may appear. The study used Harman’s one-factor test with CFA to check this bias. The results showed that the fit of the single-factor model (Chi-square/df = 7.725; GFI = 0.529; CFI = 0.764, TLI = 0.753, and RMSEA = 0.119) was far different from the proposed multi-factor model of the research framework. Therefore, if CMB exists, it does not distort the research results.

To assess the reliability of the indirect effect estimates of the paths in the structural model, this study employed a non-parametric bootstrapping procedure with 1000 resamples. Bias-corrected 95% confidence intervals were obtained by this process. For the proposed mediation paths from SQ to CS and CL via PV, and from PV to CL via CS, a bootstrapping procedure was carried out. If the 95% confidence intervals did not include zero, the indirect effect was deemed statistically significant. The results of this procedure showed that the above effect paths were statistically significant, i.e., PV has a mediating role in the research framework.

## Discussions, implications, and limitations

### Discussions

This study investigated how service quality (SQ) influences customer satisfaction (CS) and customer loyalty (CL). Furthermore, perceived value (PV) as a mediating factor between customer satisfaction (CS) and customer loyalty (CL) was also examined. Some of the key findings that were found in this study are as follows.

For the dimensions of service quality (SQ), the analysis results showed that there were relationships between the second-order latent variable SQ and other first-order latent variables, including tangible (AT), reliability (REL), responsiveness (RES), assurance (AS), empathy (EM), and safety and security (SS). These significant findings mean that these six dimensions are considered dimensions of service quality (SQ). Among them, assurance (AS) is the strongest dimension of service quality (SQ). At the same time, the newly added factor, safety and security (SS), has a factor loading no less than the other factors in the 5-factor SERVQUAL, so the results showed that SERVQUAL can be adjusted and extended to suit specific contexts. It reinforces previous studies, such as Ma [9].

Regarding the perceived value (PV) dimension, the analysis results showed that there were relationships between the second-order latent variable PV and other first-order latent variables, including functional perception (FP), experiential value (EV), and fare perception (FA). These significant findings mean that these three dimensions are considered as perceived value (PV) dimensions. In which functional perception (FP) is the strongest perceived value (PV) dimension

For the relationship between SQ and PV, the SEM analysis results showed that service quality (SQ) has a great influence on perceived value (PV). In other words, the factors of SQ, including TA, REL, EM, RES, AS, and SS, were significant in influencing PV. This finding is consistent with other research showing that perceived value is significantly impacted by service quality [58]. This result means that the perceived value of customers in taking the urban railways is significantly influenced by their perception of the service quality provided. The higher the service quality, the higher the perceived value. Simultaneously, this study stated that PV acts as an important mediator for SQ to influence CS and CL, and it was consistent with the research models in several published studies, such as Ledden et al. [37], the concept of the “indirect model” in Cronin et al. [7], a chain of SQ-PV-CS in the model’s Ma [9].

Concerning the relationship between PV and CS, CL, the analysis results showed that PV had a significant direct positive effect on CS and CL. Besides, PV had a significant indirect positive effect on CL via CS. Thus, CS and CL were the results of receiving high perceived value from the service. At the same time, the results revealed that PV was an important factor affecting CS and CL. This finding showed that customers’ satisfaction and loyalty with the urban train service increase with their perceived value. This can be explained that customers will be more satisfied with urban railways if they feel that the value provided is higher. Higher perceived value of customers will make them feel that what they receive is higher than the cost they pay, which leads to satisfaction. At the same time, customers will be more loyal to urban railways if they feel the value received is higher. The findings of this study are also consistent with previous studies (e.g., Fornell et al. [5], Bayol et al. [11], Ibrahim et al. [27], Shen et al. [28], Stuart et al. [24], Yilmaz et al. [23], Hussein and Hapsari [58], Ma [9]), reinforcing the symbiotic relationship between PV and CS, CL.

The relationship between CS and CL: The analysis confirmed that CS was positively related to CL, i.e., when CS is higher, CL increases. So, CS is key to enhancing CL. Besides, CS was also an important mediator of PV in developing CL. Several previous studies (e.g., Zhuang et al. [18], De Ona et al. [22], Lai and Chen [8], De Oña et al. [29], Yilmaz et al. [23], Nguyen-Phuoc et al. [32]) have also confirmed that there was a strong relationship between CS and CL. Therefore, the results of this study further strengthen this hypothesis.

The above analysis has shown that SQ, PV, and CS are significant in influencing CL. PV is a mediating factor between SQ and CS, CL. In other words, through its effect on PV, SQ indirectly affects CS and CL, illuminating the intricate dynamics in the relationships between service providers and customers. CS is a mediating factor in developing CL. So, service providers of urban railways must focus on improving SQ and PV to strengthen CS and CL. Simultaneously, the results showed the complex relationships between SQ, PV, CS, and CL.

### Implications

Through the results of this study, urban railway service providers, as well as managers and policy makers, have an overview and a clearer assessment of the factors (service quality and perceived value) affecting satisfaction and loyalty. From there, there are appropriate adjustments to mechanisms and policies to improve the quality of service and attract customers. Therefore, helping service providers in Hanoi of Vietnam achieve success in their business activities. Some implications of this study are described as follows: Firstly, the analysis results showed that satisfaction and loyalty of urban railway customers are influenced by their perceived value, which is in turn influenced by service quality. Therefore, suppliers need to provide good and stable quality services. This helps suppliers reduce customer care costs and is the basis for customer retention; Second, suppliers need to provide quality services as a commitment to customers. In the current competitive context of transport modes, a clear commitment to quality of service will create trust for customers of urban railways and play a key role in improving customer satisfaction as well as building long-term customer loyalty; Third, when suppliers clearly understand the aspects that customers care about regarding service quality such as tangible (AT), reliability (REL), responsiveness (RES), assurance (AS), empathy (EM), and safety and security (SS), suppliers will provide services that match customers’ expectations; Fourth, suppliers need to pay attention to improving service quality to meet the increasing needs of customers, helping them feel that the service quality is commensurate with the cost they have paid; Fifth, it can be a reference for building a set of synchronous criteria for assessing the satisfaction and loyalty of customers using urban railway services in Vietnamese cities, as well as in other cities in the world with similar conditions through a chain of Service quality (SQ) - Perceived value (PV) - Customer satisfaction (CS) - Customer loyalty (CL).

### Limitations

In addition to the above results, it is also necessary to point out the limitations of this study to provide future research directions. First, this study was implemented in a developing city with a newly operated sparse urban railway network. Hence, the generalizability of the findings here to developed cities is limited. Second, the influence on satisfaction and loyalty is not limited to the factors considered in this study, so the consideration of other factors, such as social responsibility, environmental benefits, image, etc., should be considered in further studies. Third, this study also did not compare satisfaction at different time periods because the urban railway system in Hanoi is still quite new. In the future, satisfaction assessment at different time periods should also be studied. Besides, the demographic profiles of participants should also be considered in conceptual models.

## Conclusion

The paper has investigated the impact of SQ and PV on urban railway services in Hanoi city on CS and CL. The results of the study have strengthened the theory of factors affecting CS and CL, as well as continued to confirm the mediating influence of CS. This study found that SQ indirectly affected CS and CL through its impact on PV, reflecting the complex relationship between customers and services. PV was also identified as an important mediating factor, underscoring the importance of customers assessing the benefits and costs of their urban railway service experience. Some suggestions are being made to improve long-term CL based on the study’s implications. First, to increase CS and CL, urban railway service providers should place a high priority on enhancing SQ and PV. Second, to continuously improve SQ and increase the value of customer experience, service providers of urban railways should establish a culture that highly values customer feedback. Thereby, improving operational efficiency and economic benefits. Additionally, with the discovery of the mediating role of perceived value in reinforcing satisfaction and fostering loyalty, strategically enhancing SQ and PV is crucial for studies related to urban railway services in the future.

## Supporting information

S1 File Dataset used in analysis. (CSV)
